# Robust dynamical invariants in sequential neural activity

**DOI:** 10.1038/s41598-019-44953-2

**Published:** 2019-06-21

**Authors:** Irene Elices, Rafael Levi, David Arroyo, Francisco B. Rodriguez, Pablo Varona

**Affiliations:** Grupo de Neurocomputación Biológica, Dpto. de Ingeniería Informática, Escuela Politécnica Superior, Universidad Autónoma de Madrid, 28049 Madrid, Spain

**Keywords:** Central pattern generators, Neural circuits

## Abstract

By studying different sources of temporal variability in central pattern generator (CPG) circuits, we unveil fundamental aspects of the instantaneous balance between flexibility and robustness in sequential dynamics -a property that characterizes many systems that display neural rhythms. Our analysis of the triphasic rhythm of the pyloric CPG (*Carcinus maenas*) shows strong robustness of transient dynamics in keeping not only the activation sequences but also specific cycle-by-cycle temporal relationships in the form of strong linear correlations between pivotal time intervals, i.e. dynamical invariants. The level of variability and coordination was characterized using intrinsic time references and intervals in long recordings of both regular and irregular rhythms. Out of the many possible combinations of time intervals studied, only two cycle-by-cycle dynamical invariants were identified, existing even outside steady states. While executing a neural sequence, dynamical invariants reflect constraints to optimize functionality by shaping the actual intervals in which activity emerges to build the sequence. Our results indicate that such boundaries to the adaptability arise from the interaction between the rich dynamics of neurons and connections. We suggest that invariant temporal sequence relationships could be present in other networks, including those shaping sequences of functional brain rhythms, and underlie rhythm programming and functionality.

## Introduction

Robust sequences of neural activations can be found in any nervous system, from simple invertebrate circuits^1–4^ to vertebrate systems^5–12^. As experimental and theoretical studies show, sequence generation is a key computational phenomenon to encode, control and execute information in sensory, central and motor networks^13–19^. In many cases, robust sequences underlie what is usually simply viewed and termed as a rhythm. Unveiling general principles in the generation and coordination of neural sequences, particularly in transient regimes, is an important step in relating neural activity to function and has potential impact in other fields such as rehabilitation technology, robotics and control theory^20–23^.

Central pattern generators (CPGs) are neural circuits that produce flexible rhythmic motor patterns^24,25^. Their robust and highly coordinated neuron activation sequences arise from the combination of intrinsic cell and synaptic dynamics^26,27^. Invertebrate CPGs are key neural circuits to understand sequence generation and coordination, as their cells and connections have been identified and mapped, like in the crustacean pyloric CPG that we use in this study^26,28–30^. Most CPGs have what is called a non-open topology^31^, i.e., all neurons in the CPG receive input from other cells in the circuit for transient closed-loop computation. An essential component of these sequence generator networks is reciprocal inhibition between pairs of neurons. Mutual inhibition together with electrical coupling and other non-reciprocal interactions^31,32^ underly the timing of neuron activations that shape each cycle^25,33–35^. CPGs activate muscles that produce motor rhythms and their proper function is crucial for animal survival.

**Figure 1:**
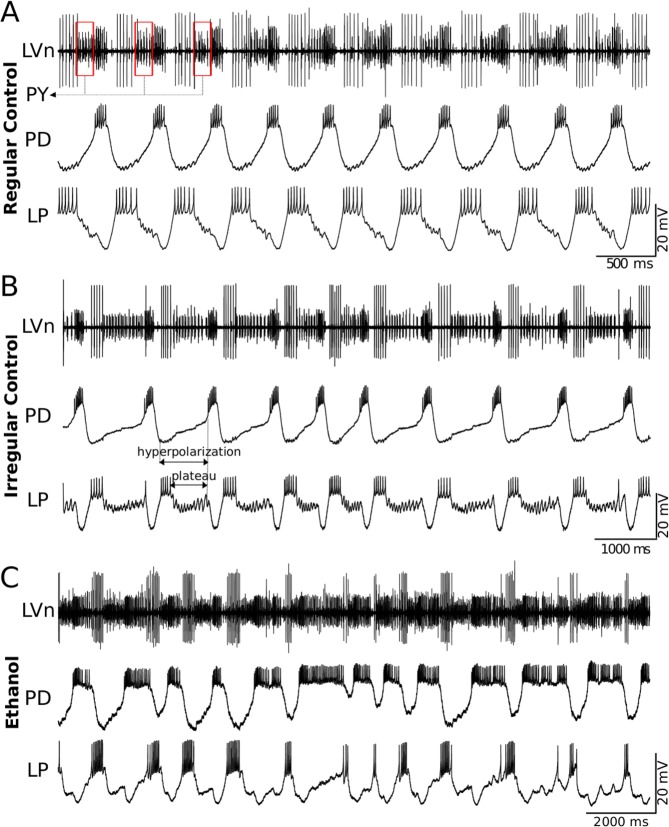
Examples of sequential activity produced by the pyloric CPG. The traces correspond to simultaneous extracellular recordings of the LVn nerve (upper trace) and intracellular recordings of PD and LP neurons in the intact CPG. Panel (A) An example of the characteristic regular triphasic spiking-bursting activity in this CPG circuit. Large spikes in the LVn trace correspond to the LP neuron. Note that LP spikes occur in antiphase with PD spikes and the respective IPSPs can be observed in the PD neuron trace. PY spikes can be observed in the extracellular recording after the LP and before the PD spikes (red boxes in the upper trace). PD and LP burst durations and hyperpolarization intervals are nearly constant in the recordings. Panel (B) Example of transient irregular spiking-bursting activity in control conditions. Note the irregular hyperpolarizations and variability in LP plateaus as compared to the regular trace shown in regular control conditions. Panel (C) Example of irregular spiking-bursting activity under ethanol (170 $mM$).

Experimental and computational studies of CPGs traditionally examine their rhythmic output from periodic spiking-bursting regimes. Recordings of crustacean pyloric CPG neurons in the semi-intact network typically show regular sequential behavior of the circuit, see^26,36^. On the other hand, *in vivo* recordings of CPG activity display further temporal characteristics and a larger degree of irregularity than *in vitro* recordings^36–38^. Recordings of isolated cells show that most of them are highly irregular^39–42^. Rich intrinsic cell and synaptic dynamics, arising from different time scales^27^, enable neurons comprising the CPG to readily establish a network rhythm in concert^26^. Recent work has proposed that variability in cycle period can be controlled by synaptic feedback^43,44^. External and intrinsic factors continuously affect the system inducing transients and therefore making them an important element of the system functionality, which can be better observed in recordings of irregular activity (see Fig. 1).

Early on, the CPG community identified the importance of phase maintenance for motor function^45–47^. Previous studies in the pyloric CPG have reported approximate maintenance of phase-frequency relationships when altering the rhythm speed by current injection^34,48–50^ or by temperature changes^36,51^. By quantifying average delays and periods and comparing across preparations, authors could show that certain elements of the rhythm maintain relative timing with changes in frequency^36,48,49^. In this context, most works discard irregular activity, as well as, transient changes. Information regarding variability cannot be ignored when characterizing the instantaneous generation and coordination of neural sequences and this information is lost under traditional average analyses.

Here, we address the changes in a cycle-by-cycle ongoing CPG rhythm, and argue that there is an instantaneous negotiation of the resultant sequence. To expose this process we included in our study irregular rhythms, i.e., activity that presented high variability within the same experiment. We considered both intrinsic variability in the preparation, and irregularity induced by ethanol. The analysis unveiled properties of the underlying robust dynamics controlling rhythm coordination, which remained unnoticed in regular rhythm regimes. In spite of the large variability seen in the experiments, we report two *dynamical invariants* in the form of strong linear correlations between pivotal time intervals that build the sequence.

Considering regular bursting activity and a dynamic-clamp protocol that altered a pyloric CPG synapse, previous work reported a dynamical invariant in which the ratio between the resulting change in average burst duration and the change in average phase lag between PD and LP neurons was tightly preserved in all preparations^52^. Here we follow this terminology, but we refer to robustly preserved instantaneous interval relationships within the variability of a preparation, as opposed to cross-preparation averaged phase maintenance. This term reflects better the transient information exchange in the circuit.

The novelty of our approach resides in the analysis of long recordings in which the variability of the activity is kept intact, i.e., we did not extract steady state regimes with nearly periodic activity from the recordings and/or average intervals among preparations as in previous studies. Rather, we characterized cycle-by-cycle variability in long intrinsic regular or irregular rhythm recordings and also in recordings under the effect of ethanol to search for intervals that sustained a robust relationship with the period and, thus, defined a dynamical invariant. Because our characterization of the rhythms shows that the intervals that build the sequence display distinct variability, it is not trivial that any of them is correlated with the period. Since connectivity is asymmetric and non-open^31^ and neurons are heterogeneous^25,32–35,53^, selecting an adequate time reference frame is crucial to expose dynamical invariants. The invariants balance flexibility and robustness of intrinsic neuron dynamics and asymmetric connectivity, in a cycle-by-cycle negotiation, which produces sequential activity even during transients. We hypothesize that dynamical invariants participate in the instantaneous coordination of the different muscles innervated by the CPG neurons, and therefore can be linked to the efficient instantaneous performance of motor activity of the system in different circumstances.

Beyond CPGs, dynamical invariants might be present in a wide variety of circuits throughout the nervous system. Frequency independent temporal ordering has been observed in different neural systems, including the hippocampus and the cortex^54,55^. The study of instantaneously preserved temporal relationships in brain rhythms can provide key insights regarding their functional role in the context of precise sequential information encoding and execution. We argue that the insight gained from examining irregular activity transients and dynamical invariants in simple CPG circuits will lead to deeper understanding of robust sequential activations in functional brain rhythms.

## Results

### Characterization of time interval variability in CPG sequential activity

The pyloric CPG of the crustacean stomatogastric nervous system presents a characteristic rhythm with three main components in a robust sequence: the Lateral Pyloric (LP) neuron, a group of six to eight pyloric neurons (PY), two electrically coupled Pyloric Dilator (PD) neurons and the Anterior Burster (AB), also electrically coupled to the PDs^24,28,56^. Panel A in Fig. 1 shows an example of extracellular recording of the LV nerve in which these three components can be clearly distinguished, along with intracellular recordings from PD and LP neurons. In control conditions, this circuit typically produces a regular and robust rhythm with nearly constant burst durations and hyperpolarization intervals (Fig. 1, Panel A).

Irregular activity can also be seen in cases of intrinsic variability in the preparation, which may be a result of external neural modulation, severed modulator nerves, etc. Panel B in Fig. 1 shows an example of extracellular (LVn) and intracellular recordings from PD and LP neurons in a stomatogastric ganglion with intrinsic variability. There are clear differences from the regular activity recordings shown in panel A: hyperpolarization intervals of both neurons are irregular and, while PD bursts remain more constant, LP presents longer plateaus and higher variability in burst duration. Irregularity can also be induced chemically, e.g. evoked by application of ethanol (Fig. 1, panel C) which is known to affect neural dynamics (see an example on rhythmic motor patterns in^57^). Pyloric rhythm under ethanol is characterized by a remarkably flexible and long PD burst duration, and variability in hyperpolarization in both neurons. At the same time, LP burst duration presents much less variability as compared to the PD neuron. Despite the large irregularity induced by ethanol, the sequence LP-PY-PD in the rhythm is still preserved. After washing or ethanol evaporation, regular activity is recovered.

**Figure 2:**
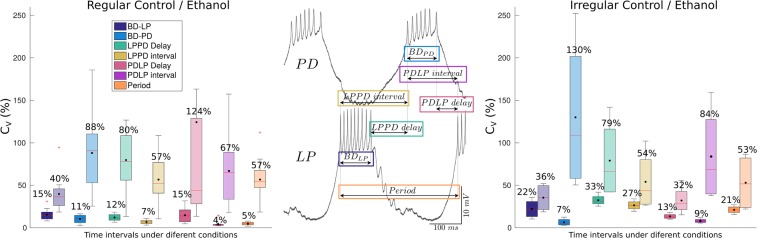
Definition and variability analysis of temporal intervals considered in this study to characterize the CPG cycle-by-cycle rhythm. Central panel: Scheme of the definition of the measured time intervals. Left and right panels: Boxplots of the coefficient of variation for the six measures in control conditions (darker color) and under the influence of ethanol (lighter hue boxes). Mean values (black dots) are displayed on top of each box. Left panel: Quantification of the variability in long recordings for preparations that were regular in control conditions ($N$ = 12). The coefficients of variation are small (4–15%) in control conditions. Under the influence of ethanol, in lighter colored boxes, there is a large increase in variability for $B{D}_{PD}$ ($\mathrm{88 \% }$), $LPPD\,delay$ ($\mathrm{80 \% }$) and $PDLP\,delay$ ($\mathrm{124 \% }$) while $B{D}_{LP}$ is more restricted in variability ($\mathrm{40 \% }$). Right panel: Intrinsically irregular preparations ($N$ = 4). One can observe an increase in variability of $LPPD\,delay$ and $LPPD\,interval$ due to the irregular hyperpolarization intervals in control conditions (see Fig. 1). After applying ethanol, there is even larger variability in $B{D}_{PD}$ ($\mathrm{130 \% }$), $PDLP\,interval$ (67–84%) and $LPPD\,delay$ ($\mathrm{79 \% }$) while $B{D}_{LP}$ variability remains more restricted ($\mathrm{36 \% }$).

The analysis of variability of CPG rhythms in all conditions was assessed by defining specific intervals with precise time references using the first and last spike of bursts from intracellular recordings. We chose seven intervals (defined in Fig. 2): $Period$, $LPPD\,delay$ (corresponding to PY neuron activity), $LPPD\,interval$, PD burst duration $B{D}_{PD}$, LP burst duration $B{D}_{LP}$, $PDLP\,delay$ and $PDLP\,interval$, and studied variability in long recordings using their coefficient of variation (${C}_{v}=\sigma /\mu \cdot \mathrm{100( \% )}$).

Note that some of these intervals are different from those used in other pyloric CPG studies that consider as time reference the beginning of the PD burst. In most studies, the PD neuron burst beginning is used as the time reference for cycle period and delays of the other so-called follower neurons^48,58,59^. A considerable variability across individual preparations was previously observed in phase-frequency relationships when the pacemaker group is used as the time reference^59,60^. Since PD neurons have strong inertia from electrical coupling among all cells in the pacemaker group, the selected time reference frame is more suitable to address the balance between flexibility and robustness, see also^49^.

Figure 2 compares the average of the coefficient of variation of the considered intervals, described in the central panel, for preparations with departing regular (left panel) and irregular (right panel) activity in control conditions and under ethanol by means of box-plots. Boxes in darker color correspond to control conditions. In regular control preparations, the values of the ${C}_{v}$ of the six intervals went from $\mathrm{4 \% }$ to $\mathrm{15 \% }$, the highest corresponding to $PDLP\,delay$ and $B{D}_{LP}$. One can observe that in control conditions variability was small but still left room for flexibility. In the case of intrinsic irregular activity, variability increased in all intervals except for $B{D}_{PD}$. Under the influence of ethanol (lighter hue boxes), both regular and irregular preparations increased the variability in all intervals. In particular, $B{D}_{PD}$ (88–130%), $LPPD\,delay$ (80–79%) and $PDLP\,interval$ (67–84%) presented a larger variability while $B{D}_{LP}$ was lower (40–36%). Note that $B{D}_{LP,PD}$ and $LPPD\,delay$ together with $PDLP\,delay$ build the triphasic rhythm. The interquartile range of the boxes indicates the variability among preparations and, in the case of ethanol, it highlights the differences of its effect on the rhythm. Overall, the system showed a wide range of variability specific to the distinct intervals that shape the rhythm with large variability in some, such as $B{D}_{PD}$, and smaller variability in others (e.g., $B{D}_{LP}$).

### Dynamical invariants

In order to identify factors shaping the CPG transient rhythm negotiation, i.e., the process of balancing flexibility and robustness of timings and sequence, we analyzed the cycle-by-cycle intervals defined above in regular and irregular rhythms. The underlying question is whether there is any property or temporal relationship in the ongoing rhythm, in addition to the sequence of neuron activations, which is preserved under different conditions (regular rhythms, intrinsic irregularity or ethanol), i.e., a *dynamical invariant*.

Departing from well-defined time references at the burst beginning and end in the LP and PD neurons, we analyzed $Period$, $LPPD\,delay$, $LPPD\,interval$, $PDLP\,delay$, $PDLP\,interval$ and burst durations $B{D}_{PD,LP}$ (see middle panel in Fig. 2), and searched for preserved correlations between pairs of intervals, even when the rhythm was very irregular. We performed this analysis cycle-by-cycle in long continuous intracellular recordings. It is important to note that most relationships between intervals were not preserved, such as $B{D}_{PD,LP}$ and $PDLP\,delay$ as a function of the $Period$, $B{D}_{LP}$ or $LPPD\,interval$ as a function of $B{D}_{PD}$ or $B{D}_{PD,LP}$ as a function of the $LPPD\,delay$ (see Table 1 columns 1–9). However, we found two relationships that presented strong linear correlations in both control an ethanol conditions: the measured $LPPD\,delay$ and $Period$ and $LPPD\,interval$ and $Period$ (Table 1 and Supplementary Figure 1). $PDLP\,interval$ presented correlation with the $Period$, however it was weaker and not consistent among preparations. Additionally, under ethanol conditions, a couple of experiments also showed correlation between PD burst duration and $Period$ (see Table 1) but it was not consistent through the rest of the experiments, as it was with both invariants. In these cases, the higher correlation with the $Period$ can be explained in terms of the very long PD bursts duration. What is unique in the intervals participating in the invariants is that the correlation exists for any interval duration category and is present in every preparation, thus we defined them as dynamical invariants. These dynamical invariants consistently remained tightly preserved with the slope of the linear regression close to one for different preparations and under different conditions.

**Table 1:** Values of the Pearson correlation coefficient $\rho$ obtained for the different combinations of instantaneous intervals considered in this study for 9 representative experiments in control and ethanol conditions (same preparations as in Figs 3 and 4).

Interval correlation	${{\boldsymbol{\rho }}}_{{\boldsymbol{Exp}}{\bf{1}}}$	${{\boldsymbol{\rho }}}_{{\boldsymbol{Exp}}{\bf{2}}}$	${{\boldsymbol{\rho }}}_{{\boldsymbol{Exp}}{\bf{3}}}$	${{\boldsymbol{\rho }}}_{{\boldsymbol{Exp}}{\bf{4}}}$	${{\boldsymbol{\rho }}}_{{\boldsymbol{Exp}}{\bf{5}}}$	${{\boldsymbol{\rho }}}_{{\boldsymbol{Exp}}{\bf{6}}}$	${{\boldsymbol{\rho }}}_{{\boldsymbol{Exp}}{\bf{7}}}$	${{\boldsymbol{\rho }}}_{{\boldsymbol{Exp}}{\bf{8}}}$	${{\boldsymbol{\rho }}}_{{\boldsymbol{Exp}}{\bf{9}}}$	t-test	${{\boldsymbol{\rho }}}_{{\boldsymbol{ < }}{\boldsymbol{Exp}}{\boldsymbol{ > }}}$
**Control**											
${\boldsymbol{LPPD}}\,{\boldsymbol{inter}}\mathrm{.[}{\boldsymbol{Period}}]$	0.976*	0.997*	0.997*	0.999*	0.997*	0.970*	0.981*	0.952*	0.988*	**1**	1.000*
${\boldsymbol{LPPD}}\,{\boldsymbol{delay}}[{\boldsymbol{Period}}]$	0.876*	0.939*	0.991*	0.988*	0.992*	0.715*	0.854*	0.392*	0.750*	**1**	0.999*
$B{D}_{PD}[Period]$	0.376*	0.587*	0.341*	0.029	−0.132	0.185*	0.108*	0.442*	0.202*	0	0.931*
$B{D}_{LP}[Period]$	−0.170*	0.352*	0.070	0.055	0.512*	0.352*	0.147*	0.135*	0.374*	0	0.924*
$B{D}_{PD}[B{D}_{LP}]$	−0.160*	0.293*	−0.090	0.279*	0.201	0.028	0.105	0.098*	0.153*	0	0.876*
$B{D}_{PD}[LPPD\,inter\mathrm{.]}$	0.342*	0.562*	0.314*	0.016	−0.154	0.147*	0.067	0.366*	0.172*	0	0.930*
$B{D}_{PD}[LPPD\,delay]$	0.354*	0.496*	0.326*	−0.024	−0.185	0.129*	0.012	0.107*	0.065	0	0.927*
$B{D}_{LP}[LPPD\,delay]$	−0.592*	0.018	−0.039	−0.092	0.424*	−0.366*	−0.357*	−0.844*	−0.315*	0	0.905*
$PDLP\,delay[Period]$	0.509*	0.355*	0.769*	0.173*	−0.417*	0.161*	0.315*	−0.110*	0.098*	0	0.929*
$PDLP\,delay[B{D}_{LP}]$	−0.105*	0.072*	−0.084	−0.006	−0.254	−0.185*	−0.030	−0.131*	−0.130*	0	0.764*
$PDLP\,inter\mathrm{.[}Period]$	0.632*	0.771*	0.787*	0.172*	−0.426*	0.375*	0.511*	0.809*	0.722*	1	0.977*
$PDLP\,inter\mathrm{.[}B{D}_{LP}]$	−0.163*	0.304*	−0.100*	0.068	−0.194	−0.203*	0.071	−0.043	0.064	0	0.843*
**Ethanol**											
${\boldsymbol{LPPD}}\,{\boldsymbol{inter}}\mathrm{.[}{\boldsymbol{Period}}]$	0.998*	0.676*	0.773*	0.866*	0.774*	0.894*	0.923*	0.979*	0.950*	**1**	0.918*
${\boldsymbol{LPPD}}\,{\boldsymbol{delay}}[{\boldsymbol{Period}}]$	0.997*	0.630*	0.743*	0.851*	0.756*	0.821*	0.910*	0.957*	0.913*	**1**	0.890*
$B{D}_{PD}[Period]$	0.395*	0.707*	0.591*	0.643*	0.934*	0.366*	0.564*	0.120*	0.531*	1	0.728
$B{D}_{LP}[Period]$	0.011	0.445*	0.386*	0.411*	0.155	0.376*	0.341*	0.505*	0.621*	1	0.548
$B{D}_{PD}[B{D}_{LP}]$	0.03	0.028	−0.039	0.151*	0.034	0.105*	0.232*	−0.091*	0.169*	0	0.335
$B{D}_{PD}[LPPD\,inter\mathrm{.]}$	0.352*	−0.011	−0.020	0.178*	0.497*	0.130*	0.204*	−0.031	0.330*	0	0.417
$B{D}_{PD}[LPPD\,delay]$	0.350*	−0.027	−0.009	0.165*	0.510*	0.089*	0.186*	−0.009	0.346*	0	0.380
$B{D}_{LP}[LPPD\,delay]$	−0.036	0.341*	0.241*	0.294*	−0.040	0.010	0.184*	0.322*	0.391*	0	0.385
$PDLP\,delay[Period]$	−0.053	0.318*	0.088*	−0.002	−0.037	0.131*	0.347*	0.711*	0.427*	0	0.601
$PDLP\,delay[B{D}_{LP}]$	−0.062	0.134*	0.055	0.057	0.229*	−0.222*	0.442*	0.306*	0.162*	0	0.112
$PDLP\,inter\mathrm{.[}Period]$	0.349*	0.785*	0.647*	0.644*	0.936*	0.591*	0.592*	0.629*	0.857*	1	0.824*
$PDLP\,inter\mathrm{.[}B{D}_{LP}]$	−0.007	0.063	−0.024	0.157*	0.042	−0.105*	0.276*	0.173*	0.292*	0	0.330

Other experiments show similar results. Regression analysis indicated that both $LPPD\,interval$ and $delay$ (bold in the table) have a strong correlation with $Period$ consistently in all the experiments. $PDLP\,interval$ present correlation with $Period$ but is not consistent among preparations, and other measured variables are not correlated. A t-test for significance of the correlation coefficients ($N$ = 16) is also included in the table. Last column represents the Pearson correlation coefficient among interval averages calculated for the 16 preparations. *Slope significantly different from 0 ($p < 8\cdot {10}^{-4}$).

Approximate phase maintenance observed in previous studies was revealed by averaging intervals across preparations (see Introduction). Following the same procedure by calculating interval averages for each preparation, correlation is found between all intervals and the averaged period and even among them in control and in most cases in ethanol conditions (see Table 1 last column). In our cycle-by-cycle analysis, strong correlation is only found between $LPPD\,interval[Period]$ and $LPPD\,delay[Period]$.

**Figure 3:**
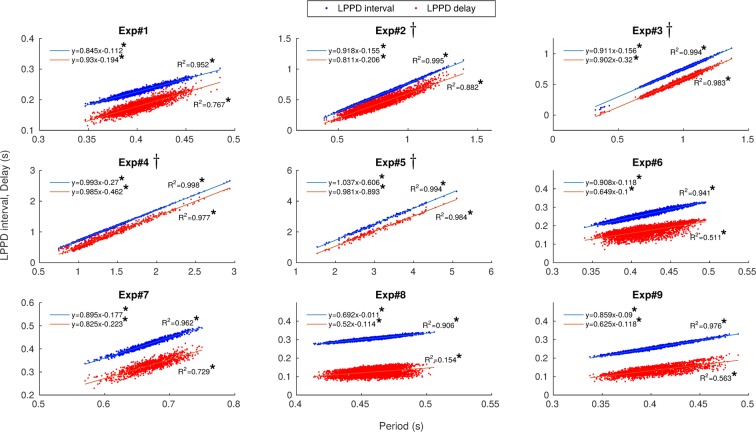
Presence of the two dynamical invariants in *control conditions* in 9 representative preparations. The correlation between $LPPD\,interval$ and $Period$ is shown in blue while the correlation between $LPPD\,delay$ and $Period$ is shown in red. Each point corresponds to one pyloric cycle of continuous recordings. Linear regressions are depicted for each experiment. Regression analysis showed that both LPPD interval and delay values increased with period. The linear dependence is indicated by ${R}^{2}$ values displayed for each experiment in the corresponding panel. ^†^Intrinsically irregular preparations. *Slope significantly different from 0 ($p < 8\cdot {10}^{-4}$).

Figure 3 depicts these two preserved relationships for 9 representative experiments in control conditions with their corresponding linear regression. The analysis includes both regular and intrinsically irregular rhythms (indicated with $\dagger$). The linear regression shows that the ratio between the change from one cycle to the next in $LPPD\,interval,delay$ and the change in $Period$ is constant. The strong linear correlations indicated the presence of these invariants despite the rhythm variability (${R}^{2} > 0.9$ for $LPPD\,interval[Period]$). We also included the special case of Exp. 8 in Fig. 3 with ${R}_{LPP\,Ddelay[Period]}^{2}=0.154$. This low coefficient of correlation can be attributed to the very small variability in control conditions resulting from a remarkably fast and highly regular rhythm in this experiment, which hides the invariant relationship. Supplementary Video illustrates, with the help of the sonification of the sequential activity, the presence of the invariants as the spiking-bursting activity progresses in time.

**Figure 4:**
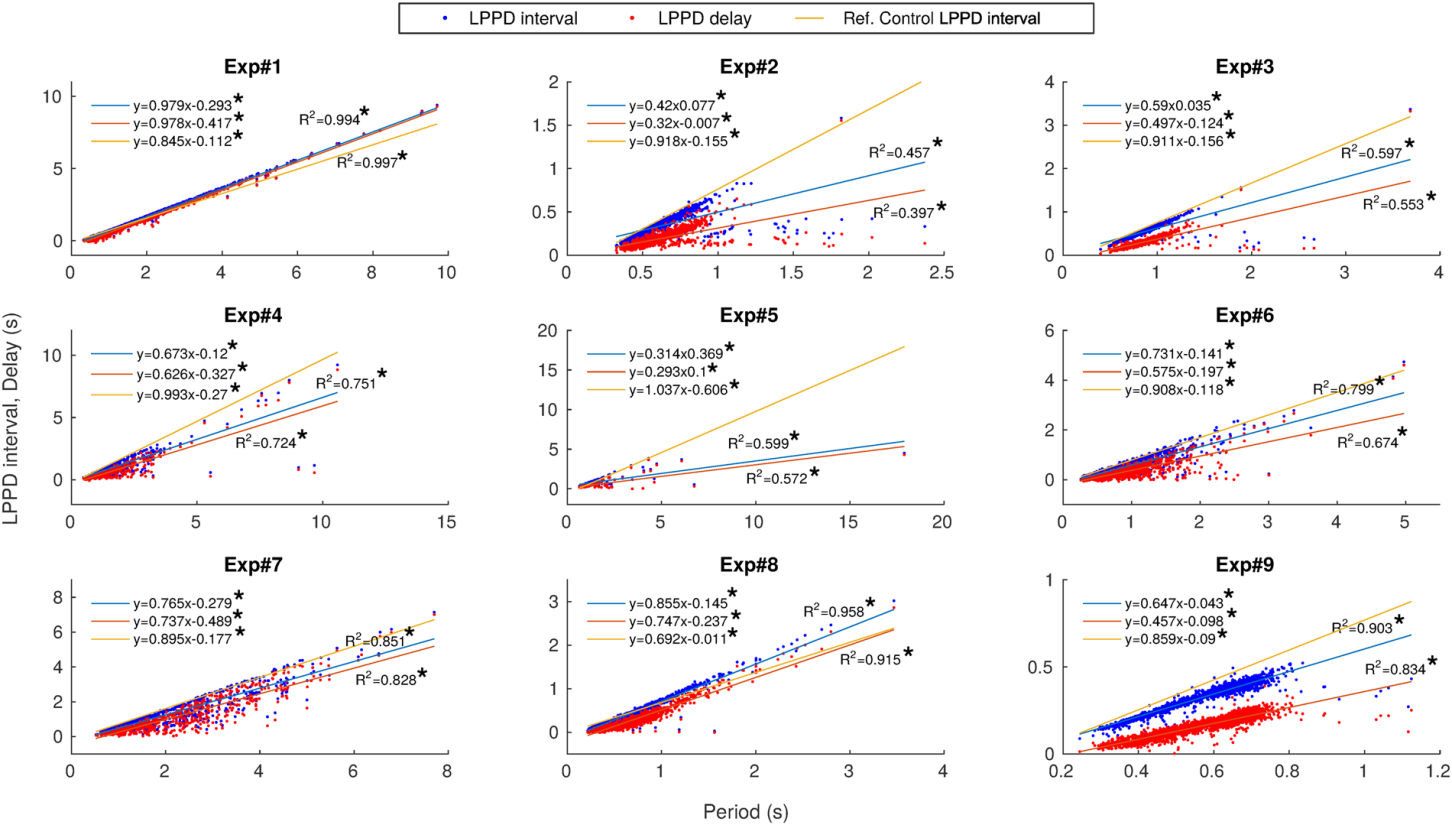
Presence of the two dynamical invariants under the influence of *ethanol* for the corresponding 9 preparations displayed in Fig. 3. The correlation between the measured $LPPD\,interval$ and $Period$ is shown in blue while the correlation between $LPPD\,delay$ and $Period$ is shown in red. Each point corresponds to one pyloric cycle. Linear regressions are depicted for each experiment. Regression analysis showed that both $LPPDinterva$l and $delay$ values increased with period. The linear dependence is indicated by ${R}^{2}$ values displayed for each experiment in the corresponding panel. Line in orange corresponds to the linear regression between $LPPD\,interval$ and $Period$ in control conditions shown in Fig. 3, and is provided to facilitate the comparison. *Slope significantly different from 0 ($p < 8\cdot {10}^{-4}$).

Figure 4 depicts these relationships for the same preparations illustrated in Fig. 3 under the influence of ethanol applied after control. Even in this condition, in which the variability of the measured intervals was very large, the invariants were still present. Note that now Exp. 8 yields ${R}^{2} > 0.9$ for both invariants. Under the influence of ethanol, the CPG rhythm can display very long bursts (lasting in some cases over 6 seconds). During some sections of the recordings in ethanol conditions, bursts in the sequence were lost. These sections that did not contain the required time references were removed from the statistics, however the percentage of dismissed bursts never exceeded 26% of the whole recording. The presence of the invariant in ethanol suggests that variability in $B{D}_{PD,LP}$ and variability in $LPPD\,delay$ compensate each other cycle-by-cycle to sustain the invariants in the rhythm.

**Figure 5:**
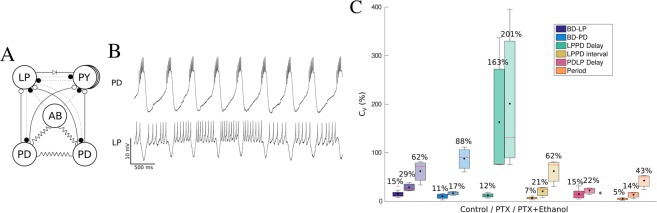
Results of blocking fast inhibitory synapses with *PTX*. Panel (A) Scheme of the connectivity of the pyloric CPG after applying picrotoxin (PTX) $5\cdot {10}^{-7}\,M$. Dotted lines correspond to blocked fast inhibitory synapses. Panel (B) Example of the spiking-bursting activity of the circuit after applying PTX. The traces correspond to simultaneous intracellular recordings of PD (upper trace) and LP (lower trace) neurons. Note that the characteristic IPSPs typical seen in the PD neuron trace are no longer present. Panel (C) Coefficient of variation (${C}_{v}$) for the six measures in three conditions: control, first column for each measure (darkest color); after applying PTX $5\cdot {10}^{-7}\,M$ ($N$ = 3), middle column; after adding ethanol to the PTX dilution ($N$ = 3), third column (lightest hue boxes). The highest variability in control conditions corresponded to $B{D}_{LP}$ and $PDLP\,delay$ $\mathrm{(15 \% )}$, while after applying PTX the highest ${C}_{v}$ corresponded to $LPPD\,delay$ with $\mathrm{163 \% }$, which is almost 14 times higher than in control. Variability in the other 5 measures also increased with PTX although more slightly. Adding ethanol to the PTX solution increased variability even further (43–201%).

One notable property of the pyloric CPG network is its asymmetric inhibitory connectivity. This connectivity could play a key role in explaining the compensation process that creates the invariants, so that, if key synapses are removed, invariants should change or even disappear. Thus, we applied picrotoxin (PTX) $5\cdot {10}^{-7}\,M$, a glutamatergic synapse blocker, which blocked the fast inhibitory synapses (see Fig. 5 panel A). Panel B in Fig. 5 shows an example of PD and LP activity after applying PTX. One can observe the irregular shape of the LP bursting activity, allowed by the low PTX concentration, the absence of LP IPSPs in the PD trace and the removal of the LP plateau. A comparison of the coefficient of variation in three conditions, control, PTX and PTX + EtOH, is shown in panel C. In control conditions, the variability was small for all measures (5–15%). After applying PTX there was a slight increase in ${C}_{v}$ for all measures except for $LPPD\,delay$ that reached $\mathrm{163 \% }$. Adding ethanol increased the variability even further (43–201%) with values similar to those obtained in experiments after applying ethanol alone (c.f. Fig. 2) except for $B{D}_{LP}$ and $LPPD\,delay$, which showed larger variability after removing the connections from the PYs and AB with PTX.

**Figure 6:**
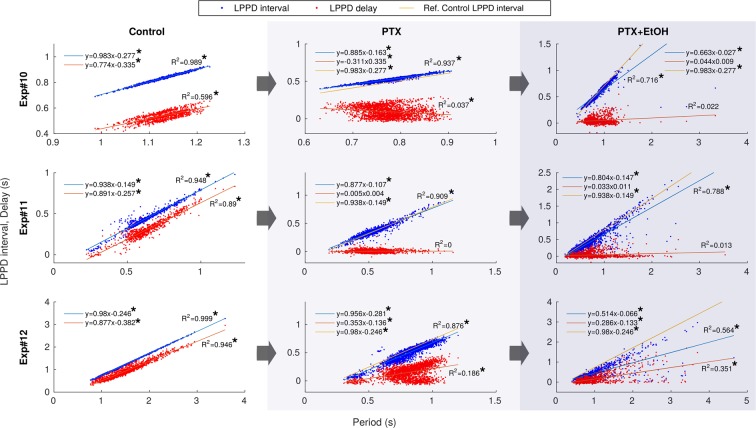
Comparison of the two dynamical invariants in three conditions: *control*, *PTX* and *PTX* + *Ethanol* in 3 different preparations. The correlation between the measured $LPPD\,interval$ and $Period$ is shown in blue while the correlation between the $LPPD\,delay$ and $Period$ is shown in red. Each point corresponds to one pyloric cycle. Regression analysis showed that only LPPD intervals increased with period. The linear dependence is indicated by ${R}^{2}$ values displayed for each experiment in the corresponding panel. *Slope significantly different from 0 ($p < 8\cdot {10}^{-4}$). Line in orange corresponds to the linear regression between the measured $LPPD\,interval$ and $Period$ in the control conditions shown in the first column.

Figure 6 shows the correlations in three experiments under three conditions: control, PTX, and PTX and ethanol. One can observe that the dynamical invariant $LPPD\,delay[Period]$ was not preserved in the absence of fast synapses with slopes tending to 0 in all experiments, as opposed to the dynamical invariant $LPPD\,interval[Period]$ that maintains a tendency similar to the corresponding control. A possible explanation for the preservation of this invariant is the LP burst duration variability, which manages to compensate PD variability, even under the effect of ethanol. More examples of the effect of PTX on the invariant correlations are provided in Suplementary Table 1 and Supplementary Figure 2.

The presence of both invariants $LPPD\,interval[Period]$ and $LPPD\,delay[Period]$ is a very robust result, observed in all control experiments performed ($N$ = 42). In the Supplementary material we also provide a script to quantify and display the two reported invariants in any simultaneous recording of LP and PD neurons.

### Time interval cycle-by-cycle analysis

**Figure 7:**
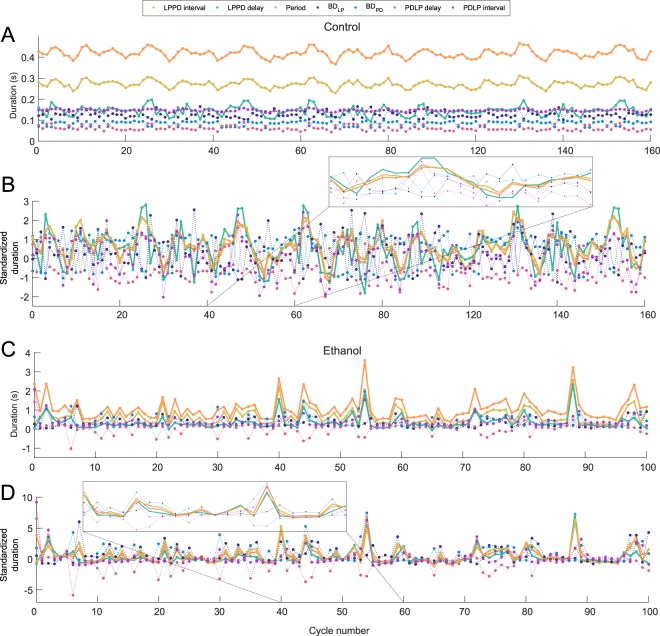
Cycle-by-cycle transient changes in the studied intervals. Panel (A), intervals $Period$, $B{D}_{LP}$, $B{D}_{PD}$, $LPPD\,delay$, $LPPD\,interval$ and $PDLP\,delay$ for each cycle. Note that despite the variability in period, $LPPD\,delay$ and $LPPD\,interval$ closely follow it. Panel (B) shows the intervals as in Panel A but with standardized duration. In this representation, the variability of all intervals are in the same range. Note that the standardized $LPPD\,delay$, $LPPD\,interval$ and $Period$, which give rise to the invariants, evolve on top of each other while the evolution of the others intertwine. Analogous representation of the cycle-by-cycle transient changes under the influence of ethanol are shown in Panels (C,D). $LPPD\,delay$ and $LPPD\,interval$ closely track $Period$ despite the induced variability. Both insets show a blow up to highlight the common evolution of the three intervals involved in the invariants (solid lines).

A major contribution of our work is the demonstration of cycle-by-cycle adjustments that give rise to the invariants, which can be indirectly be seen in Figs 3, 4 and 6. Even though in these figures each point corresponds to one pyloric cycle, the temporal relationship between points is lost in this type of representation. To better illustrate the instantaneous compensations of the different intervals in each cycle we highlight two representative example of transient changes in an intrinsically irregular preparation and under the influence of ethanol, shown in Fig. 7.

Panel A in Fig. 7 shows the evolution of each interval $Period$, $B{D}_{LP}$, $B{D}_{PD}$, $LPPD\,delay$, $LPPD\,interval$, $PDLP\,delay$ and $PDLP\,interval$ for each cycle period ($Exp\,\mathrm{\#9}$ in Fig. 3). One can observe that $LPPD\,delay$ and $LPPD\,interval$ closely follow the $Period$ despite its variability. Note that the variability in $B{D}_{LP}$, $B{D}_{PD}$ and $PDLP\,delay$ is much lower and unrelated to the $Period$. Panel B depicts all the intervals standardized so that their variability is presented in the same range. In this representation, the intervals that give rise to the invariants evolve on top of each other (see inset). However, the evolution of the intervals $B{D}_{LP}$, $B{D}_{PD}$ and $PDLP\,delay$ intertwine each other approximately compensating their variability among them. Panels C and D shows analogous cycle-by-cycle representations to A and B respectively for an illustrative experiment under the influence of ethanol ($Exp\,\mathrm{\#6}$ in Fig. 4). Note that when there is a really long PD burst, $PDLP\,delay$ interval can be negative if there is a certain overlap of the PD and LP burst. Also, in these cycles, it is likely for $B{D}_{PD}$ to be proportional to the corresponding long $Period$ (see Fig. 1). The intervals participating in the dynamical invariants, on the other hand, are correlated to the period for any interval duration category in each cycle and it is consistent in every experiment. Despite the induced large variability, $LPPD\,delay$ and $LPPD\,interval$ closely follow the $Period$.

## Discussion

Although typically characterized by frequency and synchronization, most brain rhythms throughout the nervous system are built from sequential activations of groups of neurons^12,17,61,62^. Some of these sequences are often very robust and directly related to the execution of motor commands, cognitive decisions and behavioral actions. When generating robust sequential activations, cycle-by-cycle flexibility and fine tuning of instantaneous periods, phases and event timings can be crucial for the optimization and achievement of effective functions. In this paper, we have addressed this issue in a well-known experimental model where these questions can be more easily examined.

Most experimental and computational studies on CPGs focus their analysis on regular regimes, frequently discarding non-regular transient activity^26,36^. However, the analysis of irregular CPG rhythms, rich in transient dynamics and not only in steady state activity, can unveil important properties of the robust neuron and network dynamics underlying sequence programming and coordination. Irregular rhythms were obtained in this study by two means: intrinsic irregularity, and biophysical disruption with ethanol. Moderate ethanol application did not disrupt the anti-phase relationship between LP and PD neurons, thus the robustness of the sequence was kept, but evoked variable burst duration and hyperpolarization intervals. The effect of ethanol was reversible and in most cases the neurons returned to their original rhythm after ethanol was washed out or evaporated.

As opposed to traditional regular activity recordings, irregular rhythms caused by intrinsic factors in the preparation presented high variability in hyperpolarization intervals and waveforms in both LP and PD neurons. LP presented larger plateaus and higher variability in burst duration while PD activity remained less variable. Irregularity induced by ethanol, however, presented remarkably flexible and long PD burst durations, while LP burst duration was more restricted. Ethanol also induced variability in the hyperpolarization intervals in both neurons.

Our results show that the CPG tends to preserve specific cycle-by-cycle temporal relationships between neurons even under extreme conditions, which points out to the circuit’s highly effective negotiating properties and the dynamical arrangement of the motor rhythm to balance robustness and flexibility. Using an adequate time reference frame and experimental conditions to expose transient dynamics, our characterization of cycle-by-cycle variability in CPG circuits has revealed the presence of dynamical invariants in neural sequences. Cycle-by-cycle analysis allowed to center the study of dynamical invariants in transient regimes, without losing the temporal relationship between pivotal time intervals building the sequence. Results show that $LPPD\,delay$ and $LPPD\,interval$ closely follow the changes in $Period$ despite the variability underlying both dynamical invariants. These invariants were present not only in regular control conditions but also in intrinsic irregular conditions and when high irregularity was induced by ethanol. In control conditions, the presence of both invariants was a very robust result, since they were found in all experiments performed ($n$ = 42). One plausible explanation for the invariants is that the intervals, $B{D}_{LP}$, $B{D}_{PD}$ and $PDLP\,delay$ approximately compensate their variability in each pyloric cycle. The invariant $LPPD\,interval[Period]$ is more precise than $LPPD\,delay[Period]$, this is probably because $LPPD\,interval$ contains the added variability of both $B{D}_{LP}$ and $LPPD\,delay$. It is important to note that other explored relationships among CPG activity intervals did not lead to strong correlations in the form of invariants. This might imply that they are not as relevant for the rhythm negotiation and, thus, have unrelated variability to fulfill another role. Since in our experiments we only used intracellular recordings of LP and PD neurons, we cannot discard the presence of additional preserved relationships among other neurons. CPGs could use the reported invariants and other preserved relationships to program their function under distinct circumstances, which may underlie their remarkable context-specific autonomous adaptability and functional efficiency.

It is important to emphasize that in this work we considered different time intervals from the commonly analyzed latency onset and offset, which are defined using the PD neuron first spike^36,48,49,59^. When connectivity is asymmetric, the selection of the time references to define the intervals is crucial for exposing potential dynamical invariants. LP neuron receives less connections than other neurons in the pyloric circuit. Therefore, it has more flexibility to adapt and coordinate its activity with the rest of the circuit elements in a cycle-by-cycle basis, making this neuron a better candidate as a time reference (see also^49^). Also note that the two invariants observed during cycle-by-cycle transients are different than the approximate phase maintenance reported in previous works that used cross-preparations analysis, steady activity recordings, and other time references^36,48,49,59^. Approximate phase maintenance, obtained by averaging phase and periods in different preparations or in the same preparation under different treatments^34,36,48–51,59^, might reflect some aspects of the unveiled invariants, but not their presence in cycle-by-cycle analysis, in particular during transients. In our analysis, averaging intervals within preparations (see last column in Table 1) always provided linear relationships between all intervals and the period across experiments due to all sources of interval variability canceling each other. However, only the intervals participating in the invariants are strongly correlated to the period in a cycle-by-cycle analysis in long recordings. This means that the variability of these two intervals in each cycle is restricted by a linear relation and thus results in a rule for the coordination of the sequence.

For further analysis, PTX was used along with ethanol to study how removing fast synapses in the circuit affected the invariants and tilted the interacting forces of the network that negotiate timings within a robust sequence. We observed that the specific asymmetric connectivity of the pyloric network plays a key role in shaping the invariants. After removing glutamatergic synaptic inputs by applying PTX, the correlation $LPPD\,delay[Period]$ was completely gone while $LPPD\,interval[Period]$ was still maintained. Preservation of this last invariant is probably due to the LP burst duration variability, which manages to compensate the PD variability, even under the effect of ethanol.

Most neural functions are supported by neuronal oscillatory activity, often simply referred to as a rhythm^61,63^. Rhythms are recorded in specific brain circuits, such as in CPGs, or observed in recordings spanning distinct frequencies and anatomical regions, such as the cerebellum, the hippocampus, the basal ganglia and cortical areas. In most cases, brain rhythms are characterized and quantified in regard to only their frequencies and synchronization properties. However, a wide variety of experimental works show that robust sequential activations of different neuron types participate or are recruited at different phases of the oscillations that define brain rhythms (e.g^64–70^).

Most neural rhythms, as pyloric neural oscillations, are based on inhibition as the main mechanism shaping not only the rhythmic activity^71^, but most importantly, the sequential activation of its constituent elements. Inhibition based mechanisms offer specific time windows where neurons can express their excitability, balancing the robustness of the sequence and the flexibility to tune activation timings. The actual execution of a sequential neural command, e.g., in the performance of a movement, is determined not only by the serial order of individual participants but also by their timing. This is the case for the pyloric CPG, as most likely fine timing adaptations are required to optimize the function of the motor plant beyond keeping the sequence needed to move food from one side to the other.

The unveiling of dynamical invariants in the spatio-temporal patterns of neural activity may have an important impact in robotics. Traditional robotic locomotion control paradigms are based on ad-hoc rules to deal with different scenarios (e.g. obstacle avoidance, uneven terrain, etc). The concept of dynamical invariants provides an alternative way to autonomously build constraints to drive behavior in all situations. In this context, a dynamical invariant based CPG control arising from the connectivity and rich intrinsic neuron dynamics^21^ can provide autonomous solutions to different situations informed by sensory feedback.

Beyond spiking-bursting activity and CPG function, dynamical invariants in other brain rhythms can underlie the creation of cyclic windows within oscillations when synaptic input can be most efficiently integrated for the effective execution of sequences generated in a given informational context^55,66^. We foresee that the study of specific time references and dynamical invariants in different neural systems will provide novel views on the functional role of brain rhythms and their constituent sequences.

## Methods

### Experimental design

Adult male and female shore crabs (*Carcinus maenas*) were purchased locally and maintained in a tank with 13–15 °C artificial seawater. Crabs were anesthetized by ice for 15 min before the dissection. The procedures followed the European Commission and Universidad Autónoma de Madrid animal treatment guidelines. The stomatogastric nervous system was dissected following standard procedures and pinned in a Sylgard-coated dish containing *Carcinus maenas* saline (in $mM$: 433 $NaCl$, 12 $KCl$, 12 $CaC{l}_{2}\cdot 2{H}_{2}O$, 20 $MgC{l}_{2}\cdot 6{H}_{2}O$, 10 $HEPES$, adjusted to pH 7.60 with 4 $m$ $NaOH$). After desheathing the STG, neurons were identified by their membrane potential waveforms and the spikes times in the corresponding motor nerves. Membrane potential from neurons was recorded using 3 $M$ $KCl$ filled microelectrodes (50 $M\Omega$) and a DC amplifier (ELC-03M, NPI Electronic, Hauptstrasse, Tamm, Germany). Extracellular recordings were made using stainless steel electrodes in Vaseline wells on the motor nerve and amplified with an AC amplifier neuroprobe (model 1700, A-M Systems, Bellevue, WA, USA). Data was acquired at 10 KHz using a A/D board (PCI-MIO-16E-4, National Instruments). Spike timings were obtained from intracellular recordings using Dataview (https://www.st-andrews.ac.uk/wjh/dataview/), first applying a FIR filter and then a threshold-crossing criterion to detect the beginning of each spike. Since we used a high threshold and worked with intervals, calculated by subtracting consecutive time references, the error introduced by using the beginning of the spike is mostly cancel out. In each recording, the distribution of the spikes was used to select the time windows that defined the intra and inter burst intervals, and particularly the first and last spike of each burst (see Matlab scripts in Supplementary material). The accuracy of the scripts were carefully verified for each experiment. Preparations were exposed to concentrations of (170 $mM$) Ethanol (Panreac), added directly to the bath. Glutamatergic synaptic inputs were blocked using ${10}^{-7}\,M$ picrotoxin (PTX; Sigma-Aldrich). Only preparations that completed all categories of treatment were included for this analysis.

### Statistical analyses

To analyze and quantify regular and irregular recordings, we considered several interval measures based on precise time references at the beginning and at the end of the bursts (see Fig. 2 middle panel): PD and LP burst duration $B{D}_{PD,LP}$: intervals from the first spike to the last spike of PD and LP neuron, respectively; $LPPD\,delay$: interval from the last LP spike to the first PD spike; $LPPD\,interval$: interval defined from the LP first spike to the PD first spike; $PDLP\,delay$: interval from the last PD spike to the first LP spike in the following burst; $PDLP\,interval$: interval from the first PD spike to the first LP spike in the following burst; $Period$: interval from first LP spike to the next first spike in the following LP burst. We quantified these measures in long intracellular recordings (15 min on average). There were some extreme cases in irregular rhythms induced by ethanol where time references were not well defined and the corresponding activity had to be removed from the statistics shown below. The number of bursts that had to be dismissed in these experiments ranged from $1$ to $\mathrm{17 \% }$ and $0$ to $\mathrm{27 \% }$ of the total number of bursts of LP and PD neurons, respectively, in the recordings.

The coefficient of variation defined as ${C}_{v}=\sigma /\mu \,\cdot 100\,( \% )$ depicted in the boxplots Figs 2 and 5 was calculated as an average of the ${C}_{{v}_{i}}$ of each experiment in an ensemble $N$ specified in each plot.

1 $${C}_{v}=\langle {C}_{{v}_{i}}\rangle =\langle \frac{{\sigma }_{i}}{{\mu }_{i}}\rangle \cdot \mathrm{100,}\,for\,i\in \mathrm{\{1,}\,\mathrm{2,}\,\mathrm{...,}\,N\}$$

The significance level $\alpha$ used for the null hypothesis significance test for correlations of data was adjusted according to the Bonferroni correction, which modifies the desired overall alpha level ${\alpha }_{0}$ compensating for the number of hypothesis to be tested $m$ as the following:

2 $$\alpha =\frac{{\alpha }_{0}}{m}$$

In this case, the number of hypothesis is the correlations between different combination of the defined time intervals ($m=12$). Thus, setting ${\alpha }_{0}=0.01$, the final significance level applied was $\alpha =8\cdot {10}^{-4}$.

A t-test was also performed to tested the significance of the correlation coefficients among 16 experiments for each hypothesis $m$ (see Table 1).

Standardized cycle intervals $z$ in Fig. 7 were calculated as follows:

3 $${z}_{j}=\frac{{x}_{j}-\mu }{\sigma },\,for\,each\,j\,cycle,$$

where $x$ is the interval value.

Experimental data analysis was implemented with Matlab. In the Supplementary material we provide the scripts to calculate the intervals defined in Fig. 2 from the spike-times, plot the invariants and produce barplots of the coefficient of variation. These scripts can be used for further validation in other CPG circuits and, in fact in any other candidate neural sequence.

## Supplementary information

Supplementary information

Supplementary Video

Supplementary scripts

Dataset

## Data Availability

The interval datasets that support the findings of the current study are included in this published article (and its Supplementary Information files).
