# Supplementary material for: Robust dynamical invariants in sequential neural activity
Source: Sci Rep. 2019 Jun 21;9:9048. doi: 10.1038/s41598-019-44953-2 (PMC6588702; doi:10.1038/s41598-019-44953-2)
Supplement: Supplementary file 1 — Supplementary information [file 41598_2019_44953_MOESM1_ESM.pdf]

Robust dynamical invariants in sequential neural activity

Irene Elices[1,*], **Rafael Levi**[1], **David Arroyo**[1], **Francisco B. Rodriguez**[1], **and Pablo Varona**[1,+]

[1]Grupo de Neurocomputación Biológica, Dpto. de Ingeniería Informática, Escuela Politécnica Superior, Universidad Autónoma de Madrid, 28049 Madrid, Spain
[*]irene.elices@uam.es
[+]pablo.varona@uam.es

Supplementary Information

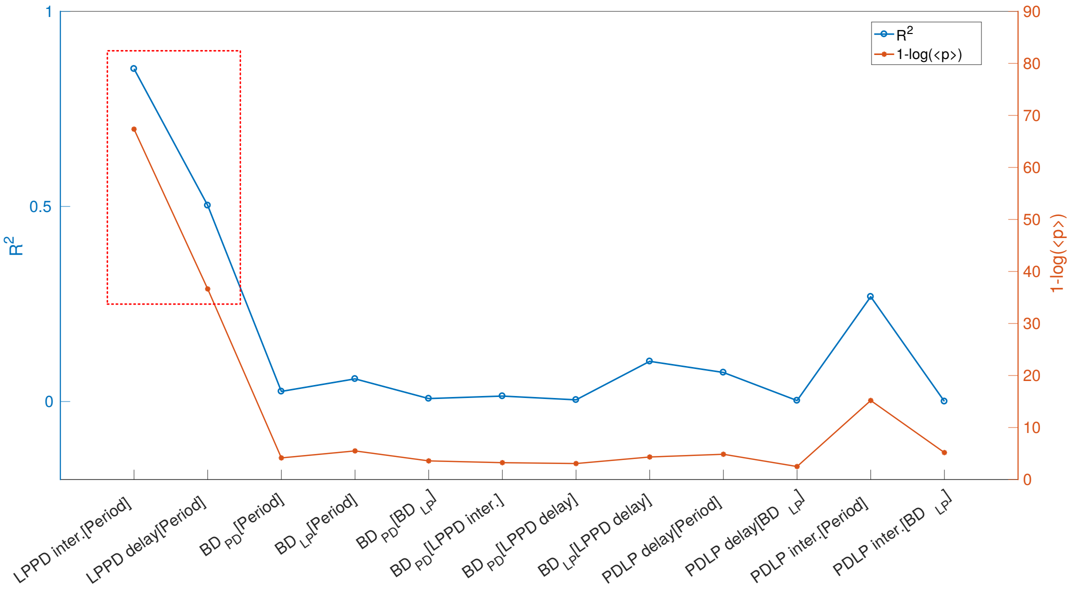

Supplementary Figure 1. Comparison of the mean correlation R^2 and P-values for the 12 combinations of time intervals shown in Table 1 (N=16). Note the larger values of R^2 in *LPPD inter.*[*Period*] and *LPPD delay*[*Period*] indicating a strong correlation. Other interval combinations yield much lower correlations as quantified by the R^2 values.

Supplementary Table 1. Values of the Pearson correlation coefficient ρ obtained for the different combinations of instantaneous intervals considered in this study for 9 representative experiments after applying PTX $5 \cdot 10^{-7}$ M (same preparations as in Supplementary Figure 2). Other experiments show similar results. A t-test for significance of the correlation coefficients is also included in the last column of the table. * Slope significantly different from 0 ($p < 8 \cdot 10^{-4}$).

**PTX**	ρ_{Exp10}	ρ_{Exp11}	ρ_{Exp12}	ρ_{Exp13}	ρ_{Exp14}	ρ_{Exp15}	ρ_{Exp16}	ρ_{Exp17}	ρ_{Exp18}	t-test
LPPD inter.[Period]	0.968*	0.953*	0.936*	0.878*	0.940*	0.483*	0.899*	0.938*	0.939*	1
LPPD delay[Period]	-0.192*	0.018	0.431*	0.036	0.750*	-0.053	0.097*	-0.092	0.195*	0
$BD_{PD}[Period]$	0.002	0.178*	0.258*	0.196*	0.698*	0.534*	0.453*	0.117	0.437*	0
$BD_{LP}[Period]$	0.585*	0.920*	0.631*	0.464*	0.310*	0.397*	0.671*	0.808*	0.480*	1
$BD_{PD}[BD_{LP}]$	0.035	0.106*	0.154*	0.078	0.261*	0.266*	0.265*	0.110	0.196*	1
BD_{PD}*[LPPD inter.]*	-0.037	0.064	0.190*	0.184*	0.629*	0.292*	0.353*	0.035	0.442*	0
BD_{PD}*[LPPD delay]*	-0.065	-0.168*	0.057	0.030	0.463*	-0.075*	0.035	-0.149	0.126*	0
BD_{LP}*[LPPD delay]*	-0.901*	-0.241*	-0.345*	-0.827*	-0.311*	-0.656*	-0.581*	-0.590*	-0.734*	1
PDLP delay[Period]	0.286*	0.319*	-0.006	0.572*	0.895*	0.303*	0.813*	-0.202*	0.519*	0
PDLP delay$[BD_{LP}]$	-0.028	0.046	-0.186*	0.118*	0.144*	-0.503*	0.444*	-0.309*	0.119*	0
PDLP inter.[Period]	-0.974*	-0.562*	-0.568*	-0.941*	-0.713*	-0.686*	-0.875*	-0.719*	-0.861*	1
PDLP inter.$[BD_{LP}]$	0.435*	0.620*	0.419*	0.241*	-0.108*	0.716*	0.156*	0.705*	0.305*	0

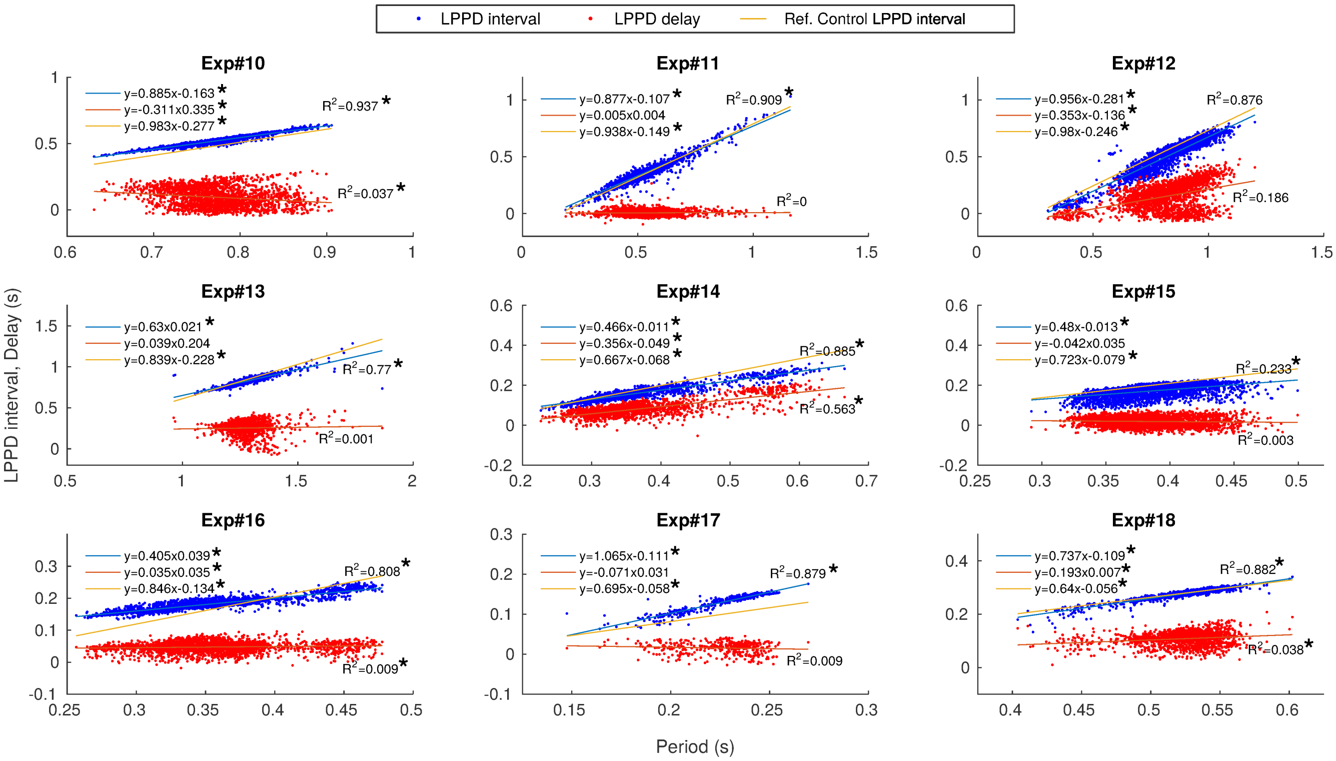

Supplementary Figure 2. Comparison of the two dynamical invariants after applying PTX $5 \cdot 10^{-7}$ M in 9 preparations. Related to Figure 6. The correlation between the measured *LPPD interval* and *Period* is shown in blue while the correlation between *LPPD delay* and *Period* is shown in red. Each point corresponds to one pyloric cycle. Linear regression is depicted for each experiment. Regression analysis showed that only LPPD intervals increased with period. The linear dependence is indicated by R^2 values displayed for each experiment in the corresponding panel. * Slope significantly different from 0 ($p < 8 \cdot 10^{-4}$). Line in orange corresponds to the linear regression between the measured *LPPD interval* and *Period* in control conditions.

Supplementary Video

Video of the evolution of the time intervals giving rise to dynamical invariants. Left panel depicts LP and PD voltage time series showing the instantaneous *LPPD interval* (blue), *LPPD delay* (red) and *Period* (black) in an illustrative experiment. Right panel shows the evolution of the dynamical invariants in time along the regression lines. Stereo sound corresponds to the sonification of LP (left ear) and PD (right ear) neurons which helps to detect the rhythm variability. The video summarizes a 27 min recording extracting representative transitions with rhythm accelerations and decelerations that entail a sudden change in $LPPD\ interval[Period]$ and $LPPD\ delay[Period]$. The video was rendered using the Python library *matplotlib* and the audio was processed using Dataview sonification tool.

Supplementary scripts

Matlab scripts that calculate the intervals defined in the text from the recording spike-times and plot the invariants and barplots of the coefficient of variation. These scripts can be used for further validation in other CPG circuits and, in fact, in any other candidate neural sequence.
